# Responses of Rice Growth to Day and Night Temperature and Relative Air Humidity—Leaf Elongation and Assimilation

**DOI:** 10.3390/plants10010134

**Published:** 2021-01-11

**Authors:** Sabine Stuerz, Folkard Asch

**Affiliations:** Institute of Agricultural Sciences in the Tropics (Hans-Ruthenberg-Institute), University of Hohenheim, 70593 Stuttgart, Germany; fa@uni-hohenheim.de

**Keywords:** diurnal growth, leaf area expansion rate, *Oryza sativa*, T_base_

## Abstract

Predictions of future crop growth and yield under a changing climate require a precise knowledge of plant responses to their environment. Since leaf growth increases the photosynthesizing area of the plant, it occupies a central position during the vegetative phase. Rice is cultivated in diverse ecological zones largely differing in temperature and relative air humidity (RH). To investigate the effects of temperature and RH during day and night on leaf growth, one variety (IR64) was grown in a growth chamber using 9 day/night regimes around the same mean temperature and RH, which were combinations of 3 temperature treatments (30/20 °C, 25/25 °C, 20/30 °C day/night temperature) and 3 RH treatments (40/90%, 65/65%, 90/40% day/night RH). Day/night leaf elongation rates (LER) were measured and compared to leaf gas exchange measurements and leaf area expansion on the plant level. While daytime LER was mainly temperature-dependent, nighttime LER was equally affected by temperature and RH and closely correlated with leaf area expansion at the plant level. We hypothesize that the same parameters increasing LER during the night also enhance leaf area expansion via shifts in partitioning to larger and thinner leaves. Further, base temperatures estimated from LERs varied with RH, emphasizing the need to take RH into consideration when modeling crop growth in response to temperature.

## 1. Introduction

Plant growth and its responses to the abiotic environment have probably been studied since humans first began to cultivate land. However, uncertainty remains on how climate change will affect the growth and yield of major crops. The difficulty arises when different plant processes respond differently to the abiotic environment, and this complexity can only be handled by models, which partly rely on assumptions. Also, abiotic factors, such as temperature, soil water availability, solar radiation, air humidity and wind, do not affect plants individually, but act in concert. A well-known example is the temperature response of photosynthesis which varies with incident light intensity and CO_2_ concentration [1]. Since weather parameters are autocorrelated under field conditions (e.g., in rainy seasons, usually, radiation and day temperatures are lower, while night temperatures and relative air humidity (RH) are higher), experiments in controlled environments are needed to disentangle the effects of individual factors. However, in order to maintain practical feasibility, the number of possible combinations of different day and night temperatures, the RH during day and night, the light intensity and duration, the CO_2_ concentration and the wind speed are limited. For greater insight into this complexity, innovative and creative experiments are required which can contribute to model improvements for better predictions of crop responses in climate change scenarios.

Leaf expansion is a major component of plant performance, enabling light capture and thus photosynthesis and biomass production [2]. In monocots, the leaves essentially grow in one dimension [3]. Therefore, to describe the rate of growth of individual leaves, leaf elongation (increase in length over time) is used instead of leaf expansion (increase in area over time). Leaf elongation rate (LER) follows a logistic function with a maximum derivative being a function of temperature [4]. Further factors influencing LER are soil water potential [5] and evaporative demand, which has been confirmed for maize and sorghum by several authors [3,6,7]. The effect of the light intensity on the LER was evident in severe shading [8,9], but not in moderate shading [3,6,7]. The effects of CO_2_ concentrations could only be observed during early development [10] or at very low CO_2_ concentrations in combination with a high vapor pressure deficit (VPD) [11].

As soil water potential and evaporative demand seem to have a larger effect on leaf elongation than light and CO_2_, plant hydraulics are a more likely candidate controlling leaf growth than carbon gains via photosynthesis. It has been argued that the expansion of individual leaves only occurs under metabolic control and, thus, is carbon limited in early stages of leaf development, whereas during later stages, LER is controlled by leaf hydraulics [2]. Another study concluded that leaf growth is regulated by plant water status at periods of high transpiration, i.e., at high evaporative demand during the day, whereas during the night, water status has no effect on leaf growth [12]. Comparing LER during day and night, higher LERs were found during the day, both under field conditions [13] and in climate chamber experiments [14]. It has been reported that in monocots the LER follows the temperature changes synchronously [15]. However, when the temperature effects are calculated using thermal time, the LER is higher at night [16].

Little is known on how differences in LER affect final leaf area, since physiological studies often focus on short-term responses without considering parameters at the plant scale [17]. Comparing different wheat species and their wild relatives, high LER was associated with higher leaf area expansion [18]. In maize, the final plant leaf area was more dependent on leaf widening, which depended on radiation, than on LER [3]. In contrast to maize, the large number of tillers produced by a rice plant adds further complexity to the relationship between individual leaf growth and plant leaf area.

The aim of this study was to investigate the effects of diurnal temperature and evaporative demand on diurnal leaf elongation rates of rice. The evaporative demand is usually given as VPD, the difference between the saturation and actual vapor pressure at a given temperature, which is generally considered as the driving force for transpiration and ultimately, the plants’ water status [19]. However, as we aim to differentiate between effects of temperature and air humidity, we used RH as the experimental factor instead of VPD, while still acknowledging the fact that both RH and temperature work as driving forces for transpiration. To relate leaf growth to carbon gains and leaf hydraulics, respectively, leaf CO_2_ exchange and stomatal conductance were measured during a 24 h period. Further, plant leaf area gains were assessed and correlated with diurnal leaf elongation rates to further advance our understanding on how short-term growth responses affect plant growth in the long-term.

## 2. Results

### 2.1. Leaf Elongation Rate

Based on a mean temperature of 25 °C and a RH of 65%, nine treatments with combinations of different day/night temperature and RH regimes were established. A “natural” temperature regime of 30/20 °C day and night temperature (Tnat), constant temperature of 25 °C (Tcon) and an inverted temperature regime of 20/30 °C (Tinv) were combined with “natural” RH of 40/90% (RHnat), constant RH of 65% (RHcon) and an inverted RH regime of 90/40% (RHinv). To distinguish effects of day/night and mean temperature, two additional temperature treatments with a constant RH of 65% were established, the first with a constant low temperature of 20 °C (Tcon-l) and the second with a constant high temperature of 30 °C (Tcon-h) (see Table 1).

**Figure 1:**
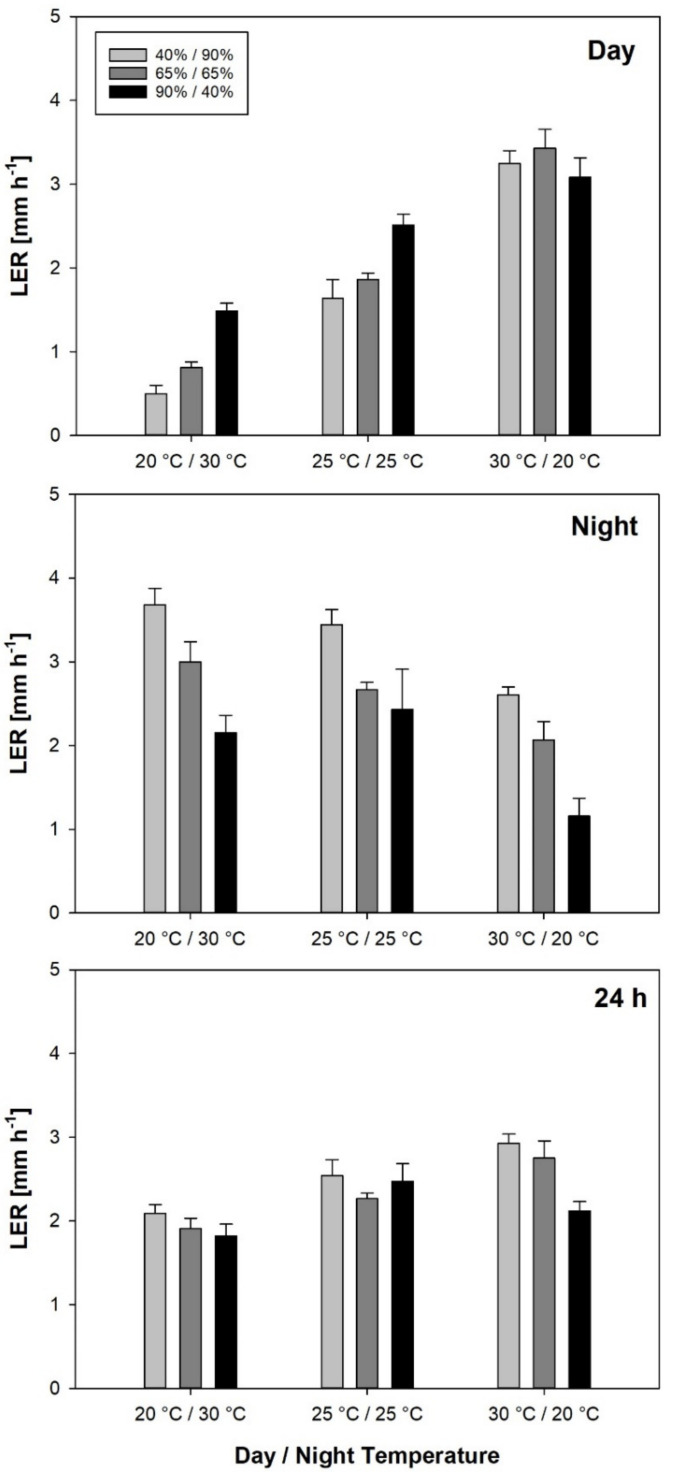
Leaf elongation rate (LER) during day, night and 24 h of plants grown at varying day and night temperatures and RH regimes. Error bars are standard errors of the mean value of 6–8 measurements.

The leaf elongation rate during the day (LER_Day_) varied between 0.50 mm h^−1^ under Tinv/RHnat and 3.43 mm h^−1^ under Tnat/RHcon (Figure 1). Temperature had a much larger effect on LER_Day_ than RH, with a statistically significant interaction of the two factors (Table 2). LER_Day_ was highest at Tnat, independent of RH, with an average of 3.25 mm h^−1^. At Tcon, LER_Day_ varied between 1.64 (RHnat) and 2.51 mm h^−1^ (RHinv), whereas at Tinv, it ranged from 0.50 (RHnat) to 1.49 mm h^−1^ (RHinv). In the observed temperature range, LER_Day_ increased linearly with day temperature at an average rate of 0.23 mm h^−1^ °C^−1^ across RH treatments. Meanwhile, low RH during the day increased the temperature effect to 0.28 mm h^−1^ °C^−1^, and high RH during the day mitigated the effect to 0.16 mm h^−1^ °C^−1^. At a constant temperature and RH, LER_Day_ ranged from 0.95 mm h^−1^ at 20 °C to 2.22 mm h^−1^ at 30 °C, but a growing temperature of 30 °C did not increase LER_Day_ significantly in comparison to 25 °C (Table 3).

The leaf elongation rate during the night (LER_Night_) varied between 1.16 mm h^−1^ under Tnat/RHinv and 3.43 mm h^−1^ under Tinv/RHcon. RH had a slightly larger effect on LER_Night_ than on the temperature, although there was not a statistically significant interaction between the two factors. LER_Night_ increased with RH during the night, from 1.85 mm h^−1^ at RHinv followed by 2.63 mm h^−1^ at RHcon to 3.20 mm h^−1^ at RHnat on average across temperature treatments. Further, LER_Night_ increased with the temperature during the night from 1.93 mm h^−1^ at Tnat followed by 2.83 mm h^−1^ at Tcon to 2.95 mm h^−1^ at Tinv on average across RH treatments without a statistically significant difference between Tcon and Tinv. At a constant temperature and RH, LER_Night_ ranged between 1.36 mm h^−1^ at 20 °C to 2.67 mm h^−1^ at 25 °C. On average across constant temperatures, LER_Night_ with 2.20 mm h^−1^ was significantly higher than LER_Day_ with 1.71 mm h^−1^ at *p* < 0.05.

The leaf elongation rate during the 24 h period (LER_24h_) varied between 1.82 mm h^−1^ under Tinv/RHinv and 2.93 mm h^−1^ at Tnat/RHnat. Temperature had a larger effect on LER_24h_ than RH with a significant interaction of the two factors. Under both RHnat and RHcon, Tnat resulted in a significantly higher LER_24h_ than Tinv. Further, under Tnat, RHnat resulted in a significantly higher LER_24h_ than RHinv. At a constant temperature and RH, LER_24h_ ranged from 1.15 mm h^−1^ at 20 °C to 2.34 mm h^−1^ at 30 °C.

The correlation of LER during day, night and 24 h in all treatments with growing conditions during day and night showed that LER_Day_ was strongly correlated with temperature during the day, whereas LER_Night_ was positively correlated with RH during the night. LER_24h_ was positively correlated with temperature during the day, but it did not show a significant correlation with RH. The correlation between LER and VPD was not significant (Table 4).

### 2.2. Gas Exchange

**Figure 2:**
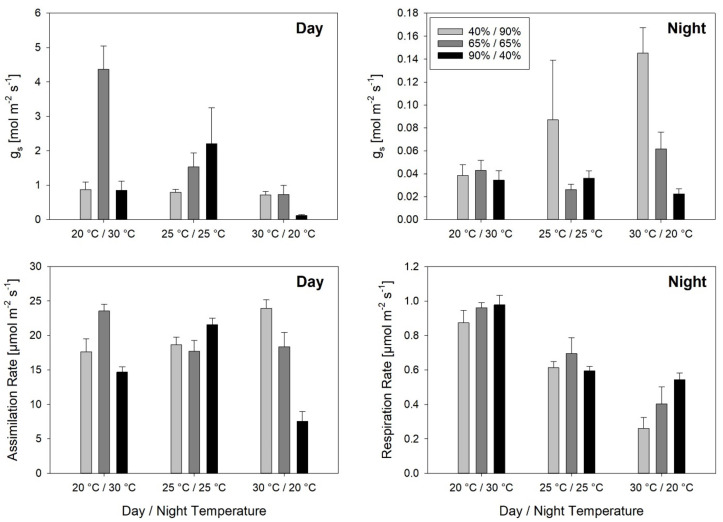
Stomatal conductance during day and night, assimilation and respiration rate of the youngest fully developed leaf of plants grown at varying day and night temperature and RH regimes. Measurements were performed during 24 h in 3–5 repetitions. Error bars are standard errors of the mean value.

Stomatal conductance (g_s_) during the day varied extremely between treatments with 0.12 mol m^−2^ s^−1^ at Tnat/RHinv and 4.37 mol m^−2^ s^−1^ at Tinv/RHinv (Figure 2) and was equally affected by temperature and RH (Table 5). However, a comparison of means showed that temperature had a significant effect on g_s_ only at RHcon, with Tinv resulting in higher g_s_ than Tcon and Tnat. Likewise, RH had a significant effect on g_s_ only at Tinv, with RHcon resulting in higher g_s_ than RHnat and RHinv. Assimilation rates (A) during the day varied between 7.5 µmol m^−2^ s^−1^ at Tnat/RHinv and 23.9 µmol m^−2^ s^−1^ at Tnat/RHnat. Temperature only had a significant effect on A at RHinv, with Tcon resulting in higher A (21.6 µmol m^−2^ s^−1^) than Tinv (14.7 µmol m^−2^ s^−1^) and Tnat (7.5 µmol m^−2^ s^−1^). RH had a significant effect on A at Tinv, with RHcon (23.6 µmol m^−2^ s^−1^), leading to higher A than RHinv and at Tnat with RHnat and RHcon, which had a significantly higher A than RHinv.

During the night, g_s_ ranged from 0.02 mol m^−2^ s^−1^ at Tnat/RHinv to 0.15 mol m^−2^ s^−1^ at Tnat/RHnat. Only RH had a significant effect on g_s_ during the night, with a g_s_ of 0.09 mol m^−2^ s^−1^ at RHnat being higher than at RHcon (0.04 mol m^−2^ s^−1^), and RHinv (0.03 mol m^−2^ s^−1^) on average across temperature treatments. The respiration rate during the night ranged from 0.26 µmol m^−2^ s^−1^ at Tnat/RHnat to 0.98 µmol m^−2^ s^−1^ at Tinv/RHinv. Temperature had a larger effect on respiration rate than RH, but no interaction between effects was observed. On average across RH treatments, respiration rate was highest at Tinv with 0.94 µmol m^−2^ s^−1^, followed by Tcon with 0.63 µmol m^−2^ s^−1^ and lowest Tnat with 0.40 µmol m^−2^ s^−1^. On average across temperature treatments, respiration rate was significantly lower at RHnat with 0.58 µmol m^−2^ s^−1^ than at RHinv and RHcon with 0.71 and 0.73 µmol m^−2^ s^−1^, respectively.

Among measured gas exchange parameters at constant temperature and RH, only the respiration rate differed between temperature treatments with a significantly higher respiration rate at 30 °C (0.96 µmol m^−2^ s^−1^) than at 20 °C (0.41 µmol m^−2^ s^−1^) (Table 6).

Correlation analysis of gas exchange parameters to temperature, RH or VPD during day and night across all treatments, showed no significant correlation of g_s_ or A measured during the day to climatic conditions (Table 7). However, g_s_ during the night was correlated with RH and VPD during both day and night, and the highest correlation coefficient was found for the positive correlation with VPD during the day. The respiration rate showed a strong and positive correlation with the night temperature.

**Figure 3:**
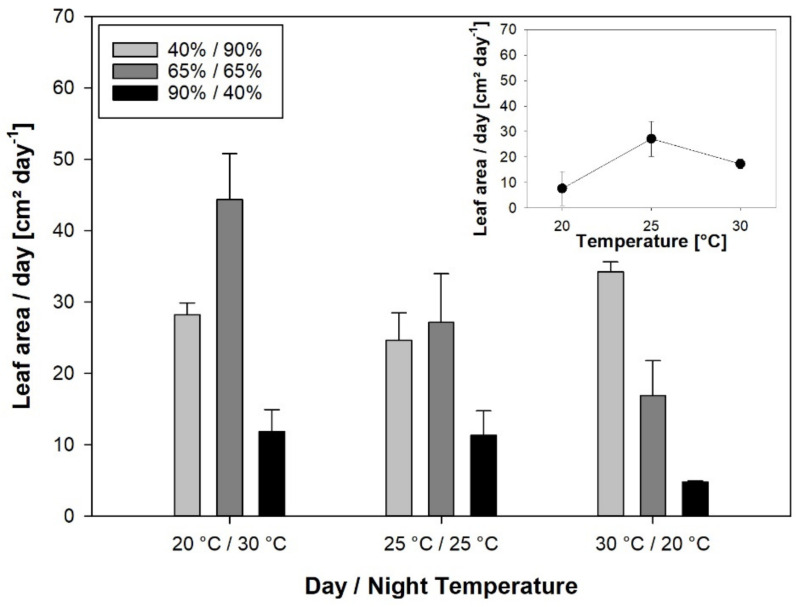
The leaf area expansion rate as leaf area increase day^−1^ plant^−1^ after two weeks of varying day and night temperature and RH regimes. Error bars are standard errors of the mean value of 3 replications.

The leaf area expansion rate, measured as the plant leaf area increase per day during the period of temperature and RH treatment, ranged from 4.8 cm^2^ day^−1^ at Tnat/RHinv to 44.4 cm^2^ day^−1^ at Tinv/RHcon (Figure 3). RH had a larger effect on leaf area expansion rate than temperature, with a significant interaction of the two effects (Table 8). Under Tinv, RHcon resulted in a higher leaf area expansion rate than RHinv with 11.9 cm^2^ day^−1^, while under Tnat, RHnat with a 34.2 cm^2^ day rate resulted in a significantly higher leaf area expansion rate than RHinv. At RHcon, the leaf area expansion rate was higher under Tinv than under Tnat with 16.9 cm^2^ day^−1^.

At a constant temperature and RH, the leaf area expansion rate ranged from 7.6 cm^2^ day^−1^ at 20 °C to 27.1 mm h^−1^ at 25 °C, without the temperature treatments having a significant effect (Figure 3, insert).

The correlation of leaf elongation rates, gas exchange parameters, and leaf area expansion rate showed a strong negative correlation between respiration during the night and LER_Day_ and LER_24h_, respectively (Table 9). Further, g_s_ during the night was positively correlated with LER_24h_. LER_Day_ and LER_Night_ were inversely correlated, while LER_24h_ was positively correlated with LER_Day_ and not correlated with LER_Night_. The leaf area expansion rate was positively correlated with A and LER_Night_.

## 3. Discussion

### 3.1. Leaf Elongation Rates during Day and Night

Daytime LER was closely correlated with daytime temperature, which had a much larger effect on LER than RH. The temperature dependence of LER has been demonstrated by several authors [4,21,22]. In pressure chamber experiments using salt-treated barley, only daytime LER could be increased, as a result it has been argued that LER was controlled by the plant’s water status during the day, but not during the night [12]. In our experiment, plants exposed to the highest evaporative demand during the light period (i.e., 2.5 kPa in Tnat/RHnat) had the second highest LER_Day_ and the highest LER_24h_ among all treatments. Thus, the positive effect of temperature on LER during the day seems to rule out the potentially negative effect of evaporative demand on plant water status. Rice has been described as an isohydric plant and shown to have a LER relatively insensitive to evaporative demand during the day [23]. By contrast, in our experiment, nighttime LER was almost equally affected by temperature and RH. High sensitivity of nighttime LER to RH has not been described so far. Since g_s_ and, thus, also transpiration, are much lower during the night even at low RH, a large effect of nighttime RH on LER seems unlikely. However, despite low nighttime transpiration rates, evaporative demand could affect leaf water potential even during the night. Negative effects of high nighttime evaporative demand have been demonstrated for the leaf water potential of wine grape [24] and the relative water content of wheat [25]. Leaf elongation rates were found to be linearly correlated with leaf water potential in soybean [26], while in ryegrass, the correlation was found only during the day, but not during the night [11]. However, studies relating daytime and nighttime leaf growth to leaf water potentials are scarce and it has been claimed very recently that more investigations are required to address the question if nighttime VPD has a negative effect on LER, given that this is a well-established observation during daytime [27].

Also, daytime and nighttime LER are not independent. Independent variation of day and night temperature showed that in rice, higher nighttime LER caused by higher night temperature was associated with a decrease in LER during the day, while LER_Night_ was not affected by higher day temperature and increased LER_Day_ [28]. In barley, artificially increased leaf growth during the day was counterbalanced by growth reductions in the following night [29]. Related to a larger variation of LER during the day, LER_24h_ was determined by LER_Day_ in our experiment. Here again, temperature had a larger effect than RH and high LER_24h_ was mainly associated with warm days and, to a lesser extent, with humid nights.

Even though the effect of RH on LER was rather weak, it should not be neglected. Leaf elongation rates have been used to calculate cardinal temperatures of crop genotypes [4,30,31], and the causal relationship between leaf elongation and leaf appearance has been discussed [32]. In our experiment, LER_Day_ linearly increased with temperature in the observed range, while LER_Night_ only slightly increased between 25 °C and 30 °C in the treatments around the same mean temperature and even decreased in the constant temperature treatments. In both cases, differences in LER_Night_ between 25 °C and 30 °C were not statistically significant. Therefore, we hypothesize that optimum temperature for LER_Night_ is below 30 °C, while for LER_Day_, the linear relationship with temperature suggests an optimum temperature above the observed temperature range. The *x*-axis intercept has been used to determine base temperatures (T_base_) from the relationship between temperature and both development rates to the flowering [33] and leaf appearance rates [34]. Although our dataset is too small to provide robust information on T_base_, taken as the *x*-axis intercept from the regression of LER_Day_ vs. day temperature, we want to highlight that the *x*-axis intercept of this regression varies with RH. While under low evaporative demand at 90% RH, tentative T_base_ is 10.6°C, which is relatively close to the T_base_ of 9.55°C described for IR64 [35], tentative T_base_ increased with evaporative demand to 17.3 °C and 18.5 °C at 65% and 40% RH, respectively. In a previous estimation of cardinal temperatures based on LER for a large panel of rice accessions at 70% RH day and night, T_base_ was found to be systematically higher than values commonly used in crop models [4]. It is clearly beyond the scope of this manuscript to provide cardinal temperatures for leaf elongation, but since precise cardinal temperatures are of such prominent importance for crop modeling, we would like to emphasize the possibility that high evaporative demand at the experimental site increases calculated T_base_.

### 3.2. Leaf Elongation Rates and Leaf Gas Exchange

Stomatal conductance responded to both temperature and RH during the day, whereas during the night, it was significantly affected by RH alone. The response to inverted temperature and RH regimes was unexpected. Extremely high g_s_ values were measured with a maximum at Tinv/RHcon, which was in the range of the calculated maximum theoretical possible stomatal conductance [36]. Measurement errors cannot be excluded here, but also in pre-experiments, where measurements resulted in elevated g_s_ at inverted day and night temperatures. High daytime g_s_ after warm nights has been observed before. In field experiments conducted in the semi-arid Sahel, noon-time g_s_ of different rice varieties was closely correlated with minimum meristem temperature, which was measured at the end of the night [37]. The mechanism behind this remains unclear. Very low g_s_ values were found at Tnat/RHinv, indicating that low RH during the night might negatively affect daytime g_s_. Effects of nighttime transpirational demand on daytime transpiration have been described for wheat [38,39]. In our experiment, unexpectedly no correlation between daytime VPD and g_s_ was found, which could be explained by the potential impact of nighttime transpiration on daytime g_s_. In summary, the measured g_s_ values suggest that stomatal responses to environmental conditions remain difficult to understand. Similarly, but with less variation, A was affected by temperature and RH treatments. Tinv at constant RH led to a relatively high A, while Tnat at RHinv led to a very low A. However, nighttime respiration was closely correlated with temperature.

No significant correlation was found between A and LER, indicating that LER was not limited by carbon assimilation. Seneweera et al. [10] found that LER at mid-tillering stage was depressed under CO_2_ enrichment and concluded that tillers were stronger carbohydrate sinks than growing leaf blades. In contrast, a high negative correlation between nighttime respiration rates and LER_24h_ was found. However, as a result of the experimental set-up it is difficult to assess if the correlation of the two parameters indicates high respiration rates depleting the carbohydrate reserves, and as a result leading to reduced LER_24h_. Alternatively, the correlation could be due to the inversion of temperature. High night temperatures lead to high respiration rates, which coincide with low LER_24h_, which is induced by low day temperatures. Results of a previous experiment in the same experimental setup suggest the first possibility. There, carbohydrate depletion after periods of increased growth and respiration during the night has been demonstrated. Whereas day temperature did not affect sucrose levels of the leaves of IR64 at the end of the day, a higher night temperature led to significantly lower sucrose concentrations at the end of the night [40].

### 3.3. Leaf Growth and Plant Growth

Unlike leaf elongation, the leaf area expansion rate was highly affected by RH, with a strong negative effect of inverted RH. In general, high air humidity has been found to have a positive effect on rice growth [41,42]. Consequently, the low leaf area expansion rate under inverted RH was a result of the low nighttime RH rather than of the high daytime RH. Although the reason for the strong negative effect of low nocturnal RH on leaf growth is still unclear, the diurnal regulation of aquaporin expression could be involved. Many aquaporins seem to be up-regulated in response to low air humidity [42], but the expression of some aquaporins increases dramatically during the day and seems to be triggered by transpiration in the presence of light [43]. As nighttime evaporative demand probably affected daytime g_s_, effects on aquaporin expression during the day seem likely. In wheat plants, high evaporative demand during the night did not affect plant dry weight or leaf area, but negatively changed the root hydraulic properties [25]. As rice has been described as sensitive to soil water deficit and evaporative demand, which are both related to its poor root system [23], changes in root hydraulic properties could have a larger effect on rice than on wheat. Under natural conditions in the field, the vapor content of the atmosphere is usually almost saturated at night, and low RH is only observed in rare cases, which means the practical implications of the effects of low nighttime RH are limited. However, studying the underlying physiological mechanism could significantly improve our understanding of plant responses in artificial environments, which will most likely increase in importance in future plant production.

Unlike LER, leaf area expansion at the plant level was significantly correlated with the assimilation rate, demonstrating the carbon limitation of expansive growth. No correlation between LER_24h_ and leaf area expansion rate was found. Instead, leaf area expansion was correlated with tiller number (data not shown), showing that tillering is more important for leaf area development than the growth of individual leaves. However, a significant correlation was found between nighttime LER and the leaf area expansion rate. Leaf growth responses could be causally related to partitioning effects because day and night temperature, and RH treatments caused large shifts in dry matter partitioning [20]. LER_Night_ was enhanced by warm and humid nights, whereas cool days and warm nights increased SLA, and high RH during the night was associated with a higher leaf mass fraction (LMF) [20]. As leaf area expansion can be described as a function of carbon gain, LMF and SLA [44], the same temperature and RH conditions that stimulate growth of individual leaves during the night also promote partitioning characteristics beneficial for leaf area expansion at the plant level.

In conclusion, the effects of nighttime RH on rice leaf growth were more pronounced than those of daytime RH. Low RH during the night has very negative effects on both the growth of individual leaves as well as the growth of overall leaf area. Since growth chamber experiments are often conducted under constant day and night RH, researchers should keep the potentially negative effect of low nighttime RH in mind. However, daytime RH also modified the growth responses to temperatures, and the results suggested an increase of T_base_ with decreasing RH. Since crop growth models are usually based on fixed cardinal temperatures, the introduction of a correction factor for RH should be considered to improve model outputs.

## 4. Materials and Methods

### 4.1. Plant Cultivation

The experiment was conducted in plant growth chambers at the Institute of Agricultural Sciences in the Tropics of the University of Hohenheim, Germany, and the experimental set-up has already been described in Stuerz and Asch, 2019 [20]. In total, 11 sets of plants were used. For each, seeds of rice variety IR64 were germinated in petri dishes on filter paper for one week. Individual seedlings were transferred into pots containing 1 L of half strength nutrient solution in the composition proposed by [45]. After another week, half-strength nutrient solution was replaced by full-strength nutrient solution and, from then onwards, full-strength nutrient solution was exchanged every week. Plants were grown in a growth chamber (Percival Intellus Environmental Controller—EA-75HIL) at 28 °C/22 °C day/night temperature, a mean temperature of 25 °C and a mean relative air humidity (RH) of 75% for the first five weeks. Afterwards, plants were transferred to another growth chamber (Percival Intellus Ultra Controller—E-75L1), where each set of plants was cultivated under different environmental conditions (Table 1) for a duration of two weeks. Temperature and RH were recorded with TinyTag TGP-4500 Dual Channel data loggers (Gemini Co., Chichester, UK) in both chambers. Day and night temperature and RH were each maintained for 12 h in accordance with day length. Artificial light was provided at a photon flux density at canopy level of about 700 µmol m^−2^ s^−1^ varying with plant height. Constant light conditions during the day resulted in a daylight integral of about 30 mol m^−2^.

### 4.2. Measurements

After an acclimation period of one week in the growth chamber, where the temperature and RH treatments were applied, measurements of leaf elongation rate and gas exchange parameters were carried out during the second week of the treatment period.

**Figure 4:**
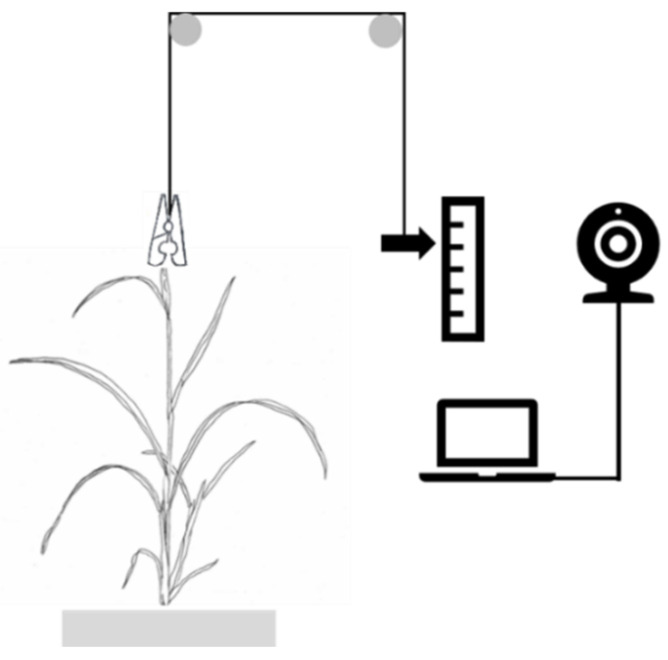
Schematic setup for leaf elongation rate measurements.

The leaf elongation rate was measured on developing leaves, during the stable phase of elongation directly following leaf appearance [46], with a visible leaf tip of 5–10 cm outside the leaf sheath of the preceding leaf. A thread was clamped to the tip of the developing leaf on one end and connected to a counterweight on the other (Figure 4). The thread was led vertically upwards via a mount to avoid leaf damage and ensure precision of the measurement. The counterweight was placed in front of a ruler. Every 30 min during 24 h, pictures of the position of the counterweight in front of the ruler were taken with a webcam. Pictures were used to determine the position of the counterweight at the onset and the end of the dark period and calculate leaf elongation rate over 24 h (LER_24h_) and during the light (LER_Day_) and the dark period (LER_Night_). During each 24 h measurement period, the developing leaves of 2 plants were assessed in parallel. These measurements were repeated on 4 consecutive days, resulting in 8 individual measurements. Since in some cases problems with the thread or counterweight impaired the measurement, between 6 and 8 repetitions of the measurement were used for each treatment.

Gas exchange was measured with a portable infrared gas analyzer for photosynthesis measurements (LCPro, ADC BioScientific Ltd., Hoddesdon, UK) on plants inside the growth chamber under ambient light, temperature, RH and CO2 conditions. The youngest fully developed leaf on the main tiller was inserted into the sample chamber and the continuous readings, which were performed during 24 h, were logged every 5 min. Measurements were repeated on a minimum of 3 consecutive days. Respiration rate was measured as assimilation rate during the night (in the absence of light) multiplied by −1.

Leaf area expansion rates were calculated as mean leaf area increase per day during the 2 weeks of the treatment period in 3 replications. For details of the leaf area measurement, see [20].

### 4.3. Data Analysis

Data analysis was carried out with STATISTICA 13 [47] using analysis of variance (ANOVA) followed by a Tukey HSD post-hoc test to analyze differences between treatments for leaf elongation rates, assimilation and respiration rates, stomatal conductance and the leaf area expansion rate. For the comparisons of temperature and humidity effects at the same mean temperature and humidity, a factorial ANOVA was used, whereas for the comparison of temperature effects in the treatments of constant temperature, a one-way ANOVA was used. Furthermore, Pearson correlation coefficients were also calculated with STATISTICA 13.

## Figures and Tables

**Table 1:** Cultivation conditions for rice plants starting 35 days after sowing until 49 days after sowing [20].

RH Day/Night [%]	T Day/Night [°C]	Treatment	
40/90	30/20	Tnat	RHnat
	25/25	Tcon	RHnat
	20/30	Tinv	RHnat
65/65	30/20	Tnat	RHcon
	25/25	Tcon	RHcon
	20/30	Tinv	RHcon
	20/20	Tcon-l	RHcon
	30/30	Tcon-h	RHcon
90/40	30/20	Tnat	RHinv
	25/25	Tcon	RHinv
	20/30	Tinv	RHinv

**Table 2:** Analysis of variance for leaf elongation rate during day, night and day & night under different day and night temperature and relative air humidity (RH) regimes around the same daily mean temperature (25 °C) and RH (65%) in 3 replications. Abbreviations: T: temperature; d.f.: degree of freedom; MS: mean square.

Time	Effect	df	MS	*p*
	T	2	29.35	<0.001
Day	RH	2	1.67	<0.001
	T × RH	4	0.92	<0.01
	T	2	6.53	<0.001
Night	RH	2	9.14	<0.001
	T × RH	4	0.26	0.58
	T	2	2.54	<0.001
Day & Night	RH	2	0.76	<0.01
	T × RH	4	0.39	<0.05

**Table 3:** Leaf elongation rate (LER) during day, night and 24 h at 3 different constant day/night temperatures and constant RH of 65%. Mean values and standard errors are given. Different letters indicate significant differences at *p* < 0.05 between temperature treatments.

Temperature [°C]	LER_Day_ [mm h^−1^]	LER_Night_ [mm h^−1^]	LER_24h_ [mm h^−1^]
20	0.95 ± 0.07 b	1.36 ± 0.10 b	1.15 ± 0.08 b
25	1.86 ± 0.08 a	2.67 ± 0.09 a	2.27 ± 0.07 a
30	2.22 ± 0.17 a	2.46 ± 0.23 a	2.34 ± 0.18 a

**Table 4:** Pearson correlation coefficients for correlations between day and night growing conditions and leaf elongations rates (LER) during day, night and 24 h, respectively, including all treatments. ***, *: significant at *p* < 0.001, *p* < 0.05, respectively. T: temperature, RH: relative air humidity, VPD: vapor pressure deficit.

Time	Growing Conditions	LER_Day_	LER_Night_	LER_24h_
	T	0.91 ***	−0.29	0.72 *
Day	RH	0.22	−0.66 *	−0.30
	VPD	0.23	0.45	0.60
	T	−0.63 *	0.60	−0.17
Night	RH	−0.22	0.66 *	0.30
	VPD	−0.04	−0.34	−0.31

*n* = 11.

**Table 5:** Analysis of variance for stomatal conductance (g_s_) and assimilation rate during day and night under different day and night temperature and RH regimes around the same daily mean temperature (25 °C) and RH (65%) in 3–5 repetitions. Abbreviations: T: temperature; d.f.: degree of freedom; MS: mean square.

Parameter	Time	Effect	df	MS	*p*
g_s_		T	2	6.78	<0.01
	Day	RH	2	6.58	<0.01
		T × RH	4	6.37	<0.01
		T	2	0.004	0.09
	Night	RH	2	0.012	<0.01
		T × RH	4	0.004	0.06
A		T	2	22.2	0.06
	Day	RH	2	115.1	<0.001
		T × RH	4	132.3	<0.001
		T	2	0.855	<0.001
	Night	RH	2	0.052	<0.05
		T × RH	4	0.026	0.146

**Table 6:** Assimilation (A) and respiration rate (R) [µmol m^−2^ s^−1^] and stomatal conductance (g_s_) [mol m^−2^ s^−1^] during day and night at 3 different constant day/night temperatures and constant RH of 65%. Mean values and standard errors are given. Different letters indicate significant differences at *p* < 0.05 between temperature treatments.

	Day		Night	
Temperature [°C]	g_s_	A	g_s_	R
20	0.88 ± 0.41	14.2 ± 3.9	0.06 ± 0.02	0.41 ± 0.12 b
25	1.53 ± 0.40	17.7 ± 1.6	0.03 ± 0.00	0.69 ± 0.09 ab
30	0.48 ± 0.22	13.6 ± 2.2	0.05 ± 0.01	0.96 ± 0.04 a

**Table 7:** Pearson correlation coefficients for correlations between day and night growing conditions and stomatal conductance (g_s_) during day and night and assimilation (A) and respiration during the night (R), respectively, including all treatments. ***, **, *: significant at *p* < 0.001, *p* < 0.01, *p* < 0.05, respectively. T: temperature, RH: relative air humidity, VPD: vapor pressure deficit.

Time	Growing Conditions	g_sDay_	g_sNight_	A_Day_	R_Night_
	T	−0.47	0.31	−0.16	−0.47
Day	RH	0.09	−0.65 *	−0.44	0.19
	VPD	−0.26	0.82 **	0.42	−0.39
	T	0.39	−0.41	0.13	0.95 ***
Night	RH	−0.09	0.65 *	0.44	−0.19
	VPD	0.25	−0.63 *	−0.26	0.55

*n* = 11.

**Table 8:** Analysis of variance for leaf area expansion rate under different day and night temperatures and RH regimes around the same daily mean temperature (25 °C) and RH (65%) in 3 replications. Abbreviations: T: temperature; d.f.: degree of freedom; MS: mean square.

Effect	df	MS	*p*
T	2	221	<0.05
RH	2	1191	<0.001
T × RH	4	237	<0.01

**Table 9:** Pearson regression coefficients for the correlations between leaf elongation rates (LER) during day, night and 24 h, stomatal conductance (g_s_) during day and night, assimilation rate (A) and respiration rate (R) and leaf area expansion rate of plants grown under different day and night temperature and RH regimes. ***, **, *: significant at *p* < 0.001, *p* < 0.01, *p* < 0.05, respectively.

	LER_Night_	LER_24h_	g_sDay_	A_Day_	g_sNight_	R_Night_	Leaf Exp. Rate
LER_Day_	−0.71 *	0.70 *	−0.46	−0.15	0.36	−0.88 **	−0.46
LER_Night_	--	0.01	0.31	0.60	0.25	0.32	0.68 *
LER_24h_	--	--	−0.34	0.40	0.75 *	−0.92 ***	0.04
g_sDay_	--	--	--	0.59	−0.20	0.47	0.60
A_Day_	--	--	--	--	0.54	−0.10	0.73 *
g_sNight_	--	--	--	--	--	−0.63	0.41
R_Night_	--	--	--	--	--	--	0.16

*n* = 9.

## Data Availability

The data presented in this study are available upon request from the corresponding author.
